# Operative *versus* conservative management for inguinal hernia: a methodology scoping review of randomized controlled trials

**DOI:** 10.1093/bjsopen/zrae116

**Published:** 2024-09-24

**Authors:** Maria Picciochi, Matthew J Lee, Samir Pathak, Jessica Banks, Jack A Helliwell, Stephen J Chapman, Neil Smart, Katy Chalmers, Sian Cousins, Natalie Blencowe

**Affiliations:** NIHR Global Surgery Unit, University of Birmingham, Birmingham, UK; Institute for Applied Health Research, University of Birmingham, Birmingham, UK; Leeds Institute of Emergency General Surgery, Leeds Teaching Hospitals NHS Trust, Leeds, UK; Division of Clinical Medicine, School of Medicine and Population Health, University of Sheffield, Sheffield, UK; Leeds Institute of Medical Research, University of Leeds, Leeds, UK; Division of Clinical Medicine, School of Medicine and Population Health, University of Sheffield, Sheffield, UK; Department of Surgery, Royal Devon and Exeter Hospital, Exeter, UK; Centre for Surgical Research, University of Bristol, Bristol, UK; Centre for Surgical Research, University of Bristol, Bristol, UK; Leeds Institute of Emergency General Surgery, Leeds Teaching Hospitals NHS Trust, Leeds, UK; Centre for Surgical Research, University of Bristol, Bristol, UK

## Abstract

**Introduction:**

There is a lack of consensus on the management of inguinal hernia with limited symptoms. To address this issue a systematic review of existing randomized clinical trials (RCTs) was performed to critically appraise all existing data on asymptomatic hernia management, focusing on generalizability.

**Methods:**

A scoping review to identify all RCTs comparing surgical and conservative management of patients with inguinal hernias was undertaken. Medline, Embase, Cochrane and ClinicalTrials.gov databases were searched. Data collected included study characteristics and definitions of population, intervention/comparator, and outcomes; and limitations of each study were also extracted. The quality and generalizability of included RCTs were evaluated using Cochrane’s ROB-2 and the PRECIS-2 tool, respectively.

**Results:**

Searches returned 661 papers; 14 full-text papers were assessed and three RCTs were identified. All RCTs included only male patients with a mean age above 55 years. All RCTs included asymptomatic patients and two included those with minimal symptoms. Different definitions for ‘minimally symptomatic’ were used in RCTs and none provided details of what was meant by conservative treatment. Follow-up periods varied between studies (1, 2, 3 years). All RCTs had an overall high risk of bias. According to PRECIS-2, two RCTs were classified as pragmatic, and one was equally pragmatic and explanatory.

**Discussion:**

This systematic review highlights a high risk of bias but a good generalizability of the findings from the RCTs conducted on minimally symptomatic inguinal hernia patients. To improve the guidelines for the management of this group of patients, more generalizable data are needed.

## Teaser

This review highlights the lack of generalizability of trials of watchful waiting for minimally symptomatic inguinal hernia. It demonstrates considerations for future trial design.

## Introduction

Inguinal hernia is a common condition affecting 27% of all men and 3% of women during their lifetime^1^. It is estimated that up to one-third of patients are asymptomatic or minimally symptomatic at the time of presentation^2^. Historically, such patients underwent surgery to prevent complications such as incarceration or strangulation, although data on the risk of these sequelae are limited^2,3^. Although a commonly performed procedure, hernia repair with mesh (using either open or minimally invasive techniques) risks short- and long-term complications including chronic groin pain (up to 12%)^4^ and recurrence (12–13% at 1 year)^5^, which may be influenced by surgeon experience and volume^6^. Given these sequelae, and the uncertainty surrounding improvements in quality of life, conservative management strategies warrant consideration in patients with no or minimal symptoms^7^. However, we must be cognisant of the increased morbidity rate conferred by emergency hernia repair should it be required^8,9^. With ongoing pressure on healthcare systems, there is an urgent need to prioritize surgical interventions that have meaningful impact on quality of life, symptomatic relief and avoidance of emergency admissions. Inguinal hernia may be a candidate for reprioritization.

International guidelines for inguinal hernia, endorsed by the British and European hernia societies, recognize watchful waiting as a potential strategy for the initial management of asymptomatic and minimally symptomatic patients^6,7^. This is also recommended by the Academy of Medical Royal Colleges in their update versionof December 2023^10^. Although the evidence supporting this recommendation includes randomized clinical trials (RCTs) and observational studies, their quality has been questioned. Previous systematic reviews found high risk of bias in three RCTs and could not directly compare outcomes as there were different definitions^11^. Considering the uncertainty associated with this recommendation, the uptake of this guidance is not widely spread^7^.

Given the limitations of the evidence on this topic, growing waiting lists particularly in high-income countries^12,13^ and the need for prioritization, an up-to-date comprehensive review is needed. This can inform clinicians on the relevance of RCTs to the populations they treat, whether current trials can inform practice and how any future work should be designed. This review summarizes and appraises current evidence comparing operative and conservative management strategies for inguinal hernia, by summarizing the key features of study design (including the PICO (population, intervention, comparator and outcome)); definitions of ‘mildly symptomatic’, ‘conservative treatment’ and ‘treatment failure’; and quality and generalizability. This will enable the identification of opportunities and gaps for further research in this area and optimize the design of downstream research.

## Methods

### Design and registration

A scoping review was conducted with reference to the Cochrane Handbook and reported in line with PRISMA-ScR guidance^14^. The PROSPERO database does not register scoping reviews.

### Eligibility criteria

RCTs comparing surgical and conservative treatment approaches involving males and/or females with any symptom severity were included. RCTs including patients with other abdominal wall hernia types were included where results for inguinal hernia patients were available separately. Non-randomized studies, systematic reviews and conference abstracts were excluded due to the high likelihood of incomplete data. RCTs where the treatment of inguinal hernia was a secondary procedure to other surgeries were also excluded. Titles and abstracts were screened independently by two reviewers. Inconsistencies were discussed and resolved by consensus with the rest of the team. The same process was applied to full-text documents.

### Information sources and searches

Systematic searches using concepts related to ‘inguinal hernia’, ‘surgery’ and ‘conservative management’ were undertaken in Medline and Embase via OVID and Cochrane databases from inception to 11 January 2023 (Supplementary Materials). Clinical trials registers (ClinicalTrials.gov, ISRCTN and ICTRP) were searched using the same time frames to capture any ongoing RCTs. Bibliographies of relevant studies and the ‘related articles’ link in PubMed were used to identify additional relevant studies.

### Selection of sources

Studies were assessed for eligibility independently by two reviewers. Where there was disagreement, this was resolved by a third reviewer. Full texts were reviewed using the same method.

### Definitions

Inguinal hernia was defined as any herniation of intra-abdominal contents or adipose tissue through the inguinal canal, including direct and indirect variations. A conservative approach was defined as any non-surgical approach (for example ‘watch and wait’ or application of a truss). Surgical repair of inguinal hernia was considered to include all potential repair techniques described in the international hernia guidelines, including different approaches (for example open or minimally invasive surgery), mode of anaesthesia (for example general or local anaesthesia) and the use of mesh or sutures to strengthen the inguinal canal.

### Data charting

Data were independently extracted by two reviewers, with any conflicts resolved with the wider team. General study characteristics, key features of study design, concept definitions and assessments of quality and generalizability were extracted for each included RCT. Where available, study protocols were also scrutinized and relevant information extracted.

#### General study characteristics

The general study characteristics including country of origin, number of included patients and centres and year of publication were recorded.

#### Key features of study design (PICO)

Characteristics of the included patients, intervention, comparator, and the primary and secondary outcomes were extracted. Where reported, constituent parts of the sample size calculation were recorded.

#### Concept definitions

Definitions for ‘minimally symptomatic’, ‘conservative treatment’ and ‘treatment failure/crossover’ were extracted verbatim. Any definition of, and rationale for, crossover was also extracted, as well as the rate and follow-up time.

#### Quality assurance

Reporting of any standardization of intervention delivery (for example approach, key steps, mesh fixation, and who delivered surgical interventions) was extracted. To provide contextual information about study setting, details about hospital and surgeon volume and expertise were also extracted.

### Critical appraisal

Two forms of appraisal were performed. First, risk of bias (ROB) assessment was performed using the Cochrane ROB tool^15^. Generalizability was assessed using the PRECIS-2 tool (PRagmatic Explanatory Continuum Indicator Summary)^16^. This is designed to aid trialists in understanding how pragmatic or explanatory an RCT is. Each trial was scored from 1 (very explanatory) to 5 (very pragmatic) for nine domains (Supplementary Materials, *Fig. S1*). A more pragmatic trial reflects what would be expected to happen in the ‘real world’ whereas explanatory trials tend to take place in an idealized setting, making it difficult to extrapolate results to other settings. Recent recommendations for retrospective use of the PRECIS-2 tool suggest that where there is insufficient information to complete a domain, they should be left blank. Disagreements were resolved by consensus.

### Synthesis of results

Data were analysed and presented descriptively using means, proportions and rates, where applicable. ROB-2 evaluation was presented using the tool algorithm, where each domain was classified as ‘low risk of bias’, ‘some concerns’ or ‘high risk of bias’. PRECIS scores were presented using ‘wheels’ as recommended by the authors^16^. In line with the study objectives, meta-analysis was not performed.

## Results

### Search results

**Fig. 1:**
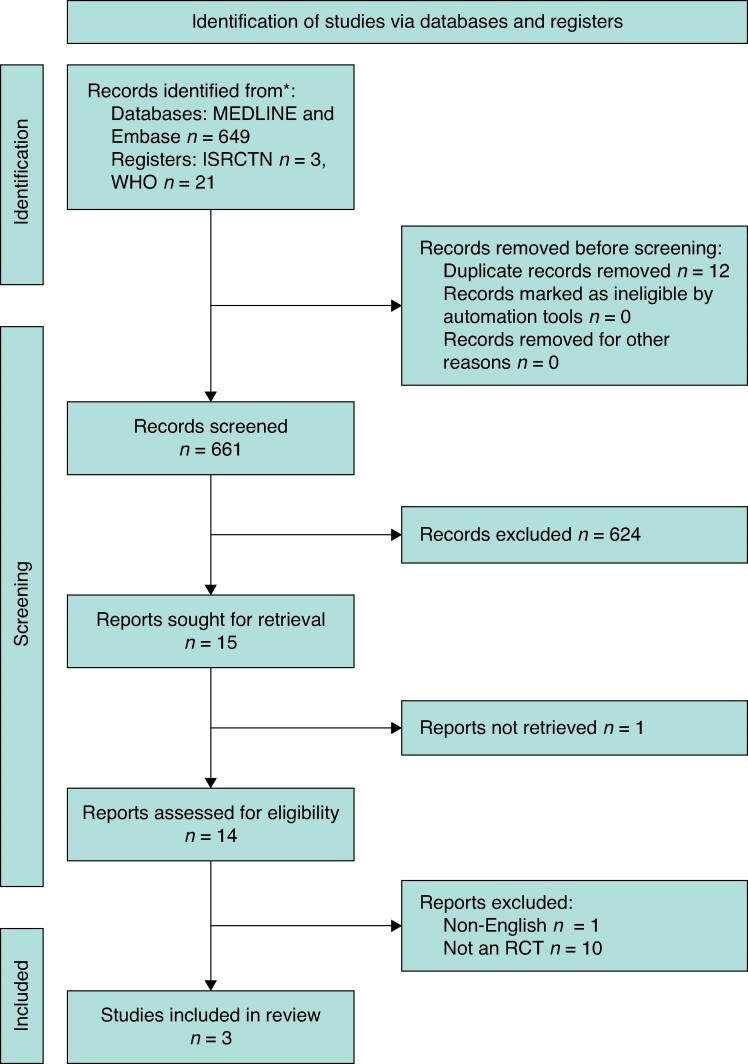
**PRISMA flowchart of included studies** This figure shows the identified and selected studies during this review. ICRT, International Clinical Trials Registry Platform; WHO, World Health Organization; RCT, randomized clinical trial.

A total of 673 records were identified (databases = 649, trials registries = 24). Of these, 661 records were screened and 14 full texts reviewed, with three studies ultimately included (*Fig. 1*).

### General study characteristics

Included RCTs were conducted in the Netherlands and Belgium^17^, UK^18^, and USA and Canada^19^ and published after the year 2000, with a total of 1376 patients. All RCTs were multicentre^17–19^.

### Key features of study design

Key PICO characteristics are presented in *Table 1*. The minimum age for recruitment into trials was 18 years^19^, 50 years^17^ or 55 years old^18^. The mean age of included patients was always above 55 years (57.5^19^, 65.1^17^ and 71.4^18^ years). All RCTs included asymptomatic patients, and two also included those with minimal symptoms^17,19^. Symptom severity was assessed using a visual analogue pain scale^18^ and a 4-point pain/discomfort score^17–19^.

**Table 1:** Summary of key features of study design (PICO)

First author, year	Setting	Inclusion criteria	Exclusion criteria	Details of surgical procedure	Details of watchful waiting	Primary outcome	Primary outcome timing	Verbatim conclusion
O'Dwyer, 2006	Multicentre (UK, *n* = 160)	Male ≧55 years Asymptomatic	Pain on examination or at rest Unfit for local anaesthetic repair Irreducible hernia	Tension-free mesh repair under local or general anaesthetic	Telephone number to contact if pain worsened	Pain and general health status (SF-36) at 12 months	1 year	‘Repair of an asymptomatic inguinal hernia does not affect the rate of long-term chronic pain and may be beneficial to patients in improving overall health and reducing potentially serious morbidity.’
Fitzgibbons, 2006	Multicentre (USA and Canada, *n* = 720)	Male ≧ 18 years Asymptomatic or minimally symptomatic	Pain limiting usual activities Difficulty in reducing the hernia Undetectable hernias Hernia repair within last 6 weeks Local or systemic infection ASA > 3 Participation in other clinical trials	Standard Lichtenstein open tension-free repair	Written instruction on activity, diet, pain, sexual activity, avoidance of constipation recognition of hernia complication	Pain and discomfort interfering with usual activities	2 years	‘Watchful waiting is an acceptable option for men with minimally symptomatic inguinal hernias. Delaying surgical repair until symptoms increase is safe because acute hernia incarcerations occur rarely.’
de Goede, 2018	Multicentre (Netherlands and Belgium, *n* = 496)	Male ≧50 years Asymptomatic or minimally symptomatic First recurrence	Bilateral or scrotal or femoral hernias ASA 4	Surgical technique defined by the surgeon	Written instruction on recognition of hernia complication	Pain	2 years	‘Our data could not rule out a relevant difference in favour of elective repair with regard to the primary endpoint. Nevertheless, in view of all other findings, we feel that our results justify watchful waiting as a reasonable alternative compared with surgery in men aged 50 years and older.’

PICO, Population, Intervention, Comparison, and Outcome; SF-36, Short Form Health Survey; ASA, American Society of Anesthesiologists.

The type of hernia repair offered to patients in the intervention groups was open mesh repair in two trials^18,19^ and surgeons’ choice in the third^17^.

Watchful waiting was a variably defined intervention. de Goede *et al.* provided written instructions to aid recognition of a hernia complication such as incarceration or strangulation, and seek help accordingly^17^. O’Dwyer *et al.* delivered watchful waiting with a focus on hernia pain or complications. Participants were given a telephone number to contact should worsening symptoms or complications occur. Participants were seen face to face at 6 months, 12 months and yearly thereafter^18^. Fitzgibbons *et al.* delivered it by providing patients with instructions around activity, diet, pain management, sexual activity and avoidance of constipation. They were also informed of warning symptoms of complications and told to contact a physician if problems developed. They were reviewed face to face in line with trial protocols^19^.

The primary outcome was different across the three RCTs: pain at 2 years^17^, and pain and general health status at 1 year^18^ and at 2 years^19^. There was inconsistency in patient-reported outcome reporting. Pain was assessed using different scales (visual analogue scale (VAS) *versus* 4-point pain/discomfort score^17,19^) and general health status was evaluated with SF-36 in all three studies, and EQ5D in one UK-based study^18^. Outcomes for the watchful waiting group included events of acute incarceration and crossover to surgery. Surgical complications were well characterized, with 21 unique outcomes reported (Supplementary Materials, *Table S1*).

### Concept definitions

‘Minimally symptomatic’ was defined as ‘mild pain without limiting the usual activities’ in the two RCTs where this group of patients was included^17,19^ (*Table 2*).

**Table 2:** Definitions presented in each study

First author, year	Minimally symptomatic	Treatment failure/Crossover	Follow-up
O'Dwyer, 2006	NA*	‘Patient in observation arm that required operation—hernia acutely irreducible, pain or increase in size such that interfered with work or leisure activities.’	Three face-to-face assessments with physical examination and completion of scales to assess pain and general health status: baseline, 6 months and 12 months
Fitzgibbons, 2006	Absence of hernia-related pain or discomfort limiting usual activities	Absent	Four assessments with physical examination: baseline, 6 months, 1 year, 2 years
de Goede, 2018	‘0 or 1 on a 4-point pain/discomfort score.’†	Absent	Five assessments with physical examination: baseline, 3 months, 1 year, 2 years, 3 years

^*^NA—not applicable considering this randomized clinical trial only included asymptomatic patients. †Fitzgibbons score: 0—No pain or discomfort due to the hernia when working, exercising or performing any of a patient’s usual activities; 1—Mild pain or discomfort due to the hernia when working and exercising that does not prevent a patient from performing his usual activities; 2—Moderate pain or discomfort due to the hernia when working, exercising, and performing any of a patient’s usual activities; 3—Severe pain or discomfort due to the hernia when working, exercising, and performing any of a patient’s usual activities.

‘Treatment failure/Crossover’ of watchful waiting was defined as patients being in the conservative treatment arm needing surgery, whether it was caused by a complication or patients describing worsening of pain. In all RCTs patients were advised to seek attention if a change in symptoms was recognized. The number of predefined assessments that included physical examination was different across all RCTs.

The crossover rates were measured at different time points across the studies: 15 months^18^, 3 years^17^ and 4 years^19^. The main indication for crossover for surgical repair was an increase in pain as shown by two RCTs that described reasons for crossover, although the extent of this was not reported^18,19^.

### Quality assurance

There was a lack of homogeneity in the surgical approach and the surgical technique chosen across all three RCTs, with a lack of information related to the procedure itself, such as mesh used and suture to fix the mesh. Additionally, no information was provided that related to who was performing the repair or how much experience the surgeon had. Centre volume in the hospitals where the RCTs occurred was also not reported.

### Critical appraisal and synthesis of results

All three RCTs were considered at overall high risk of bias, as evaluated by the RoB-2 tool and its six domains (*Table 3*). Outcome measurement was the only domain where all RCTs were considered at high risk of bias, whereas missing outcome data was the only domain with low risk of bias across all RCTs. There was only one RCT that had low risk of bias regarding the deviations from intended intervention domain.

**Table 3:** Risk of bias assessments

	Domains					
Author (primary outcome)	Randomization process	Deviations from intended intervention	Missing outcome data	Measurement of the outcome	Selection of the reported result	Overall bias
Fitzgibbons (pain)	L	H	L	H	S	H
de Goede (pain)	L	H	L	H	S	H
O’Dwyer (pain)	S	L	L	H	S	H

L, low risk of bias; S, some concerns; H, high risk of bias.

**Fig. 2:**
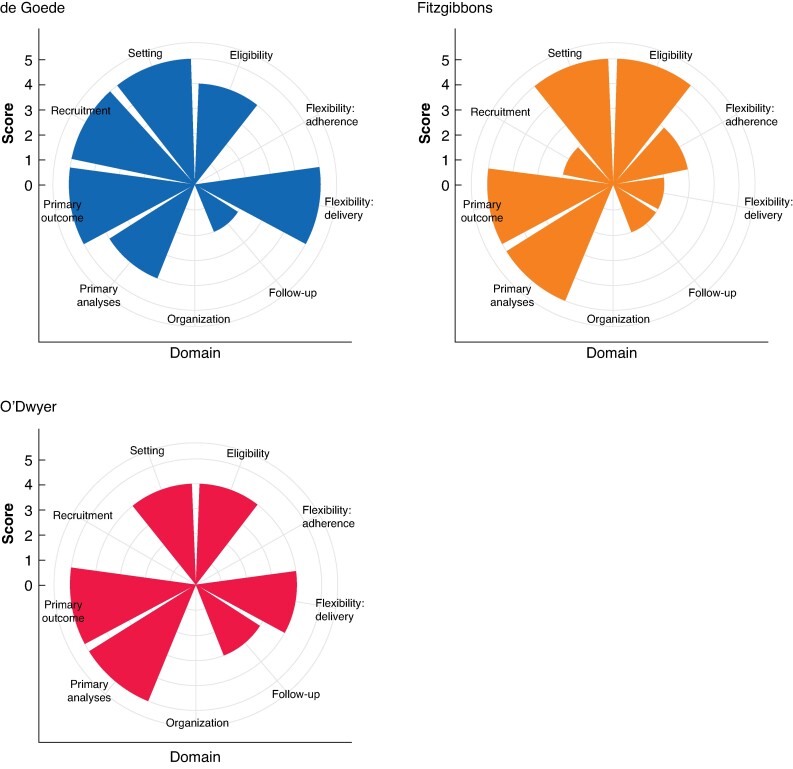
**Evaluation of each study with PRECIS wheels** This figure shows the application of PRECIS-2 wheels to each included study. This also evaluates nine domains that are related to trial design decisions and include: Eligibility criteria, Recruitment, Setting, Organization, Flexibility delivery, Flexibility adherence, Follow-up, Primary outcome, Primary analysis. PRECIS, Pragmatic Explanatory Continuum Indicator Summary. Trials that take an explanatory approach produce wheels nearer the hub; those with a pragmatic approach are closer to the rim.

PRECIS wheels are displayed in *Fig. 2* and tabulated in Supplementary Materials, *Fig. S1*. Overall, all three RCTs were more pragmatic than explanatory when measuring all domains and summing scores (mean score 3.6^19^  *versus* 4.3^17^  *versus* 4.2^18^). The only domain that was more explanatory than pragmatic was follow-up, with a mean score of 2.3. This was due to a more intensive follow-up regime and additional tests. It was not possible to derive PRECIS scores for organization and flexibility (adherence) domains due to an absence of information.

## Discussion

This study has demonstrated that the three multicentre RCTs comparing conservative *versus* surgical management of asymptomatic or minimally symptomatic inguinal hernia patients had a high risk of bias, translating a lowinternal validity. This is largely concordant with the findings from previous reviews^11^; however, it adds value through thorough interrogation of intervention definitions and assessment of designs with PRECIS-2.

The review highlights several methodological challenges when interpreting the included studies. First, there are challenges around the definition of symptom burden, considering the different definitions used. This was largely based on VAS pain scores or unvalidated assessment tools, for example the latterly named ‘Fitzgibbon score’^19^. These are unidimensional and typically relate to pain only. Hernia symptoms are multidimensional, and such an assessment is unidimensional. Where this subjective judgement is used as an inclusion criterion for a trial, it raises some concerns as this is a gameable assessment and may lead to unintended selection bias. Although the multidimensional impact of hernia symptoms was assessed using SF-36 in all studies, this is a generic quality-of-life tool, not disease-specific. There is likely to be value in developing an inguinal hernia–specific patient-reported outcome measure (PROM) to inform future studies.

Watchful waiting was an inconsistently defined intervention. Although there was general advice on seeking help should incarceration or strangulation occur, there was variation in other aspects of the strategy. Patients received face-to-face follow-up, which may be difficult to deliver in a stretched health system, especially when intervention is unlikely. Fitzgibbon *et al.* provided general holistic advice on avoiding problems with the hernia^19^. It is not clear if these additional steps provided reassurance to patients, impacting help-seeking for relatively minor symptoms. As the majority of healthcare consultations take place in primary care^20^, implementation of a watchful waiting approach would require engagement of general practitioners. This would include access to guidance on when to refer, safety nettings and easy return access to surgeons.

There was relative inconsistency in time horizons for follow-up, with studies covering different periods beyond 18 months. The natural history of an inguinal hernia is poorly understood; therefore, it is not clear what time frame is appropriate to establish safety of a non-operative strategy. Some previous studies have shown that mandating a non-operative strategy results in an increase in emergency presentations of hernia^21,22^. A follow-up study by Van der Dop *et al.*, published after this review was completed, found that at 12 years of follow-up there was a 3.9% incarceration rate in the watchful waiting group, and more than half of the patients in this group crossed over to surgery^23^.

The generalizability of these studies is significantly limited by the trial population. Although two of the studies included an age threshold for inclusion in the fifth decade, one study permitted inclusion from age 18. Despite this, the included population skewed towards the sixth or seventh decade of life for the studies. This means that findings cannot be extrapolated to those of working age. It is important to note that pain can have different impacts on activities of daily living, influenced by expectations of health status^24,25^. This means that the symptom burden experienced by participants in these studies may differ from the general population due to differences in age, co-morbidity, work and activities of daily living. Aside from this, the more notable limit on generalizability comes from the exclusion of women, who account for around 1 in 10 inguinal hernia presentations^26^. European Hernia Society guidance does not advocate watchful waiting in groin hernia for women, partly due to the risk of incorrectly diagnosing a femoral hernia^7^. Given the variation in eligible population, aspects of watchful waiting and outcome measurement, it may not be appropriate to pool data from these trials. This was a significant limitation also identified in the previous systematic reviews, especially when an attempt to combine the data from the different RCTs was made^11^. It is clear that standardization of PICOs is required for future studies.

Outcome measurement is a challenge in studies comparing operative with non-operative outcomes. Traditionally favoured assessments such as complications cannot be matched across the two trial arms/groups. Studies were relatively consistent in reporting pain and generalized quality of life across all groups. Reporting of surgical outcomes was variable, and likely requires some additional consideration to standardize reporting. This may take the form of a core outcome set^27^. Similarly, reporting of operative or non-operative interventions should be addressed with appropriate frameworks^28,29^.

There is perhaps a more fundamental consideration in study design: whether crossover to surgery reflects ‘failure’ of watchful waiting. Patients may be satisfied with an outcome of deferred surgery, meaning this is not a failure. This refers back to the need for multidimensional assessment of hernia symptoms and patient-centred outcomes. There may also be an issue around stratification of patients. The COVID-19 pandemic forced a natural experiment of watch and wait for inguinal hernia. This found low rates of emergency surgery (∼5%) at 65 months of follow-up^30^. This may suggest that there is a subgroup of patients where watch and wait would be safe. Understanding the characteristics of those who crossed over to surgery might help better stratify future care and research.

This review is limited by the number and quality of papers included. However, it provides a robust assessment through a methodological lens, using validated tools. Best practice was followed including searching of multiple databases and dual review and extraction.

The findings of this study demonstrate that the current evidence is only applicable to older men, albeit with several caveats. Specifically, watchful waiting is a poorly defined intervention of uncertain duration; minimally symptomatic hernia is not adequately defined and a highly subjective definition; outcomes do not necessarily reflect patient priorities. Should policy makers wish to consider implementation of such a strategy, then consideration should be given for exemptions based on risk of strangulation and ensuring that it applies to only the older patient group, which is currently not mentioned in the most updated recommendations^6^. There is a need for assessment of the impact of watchful waiting across all ages and both sexes, using a disease-specific PROM. Furthermore, downstream system impacts of watchful waiting should be monitored, including potential increased rates of emergency hernia surgery^22^. Given the challenges related to crossover, it may be that a traditional RCT is not the optimum design to influence this. Lessons could be taken from similar clinical problems that have used cohort studies, decision aids and cluster randomized studies^31^. This would also allow identification of those at highest risk of requiring surgery. This should be supported by the implementation of a core outcome set.

## Supplementary Material

zrae116_Supplementary_Data

## Data Availability

The data underlying this article will be shared on reasonable request to the corresponding author.
