# Correction to: Operative *versus* conservative management for inguinal hernia: a methodology scoping review of randomized controlled trials

**DOI:** 10.1093/bjsopen/zrae164

**Published:** 2025-01-24

This is a correction to: Maria Picciochi, Matthew J Lee, Samir Pathak, Jessica Banks, Jack A Helliwell, Stephen J Chapman, Neil Smart, Katy Chalmers, Sian Cousins, Natalie Blencowe, Operative *versus* conservative management for inguinal hernia: a methodology scoping review of randomized controlled trials, *BJS Open*, Volume 8, Issue 5, October 2024, zrae116, https://doi.org/10.1093/bjsopen/zrae116

In the originally published version of this manuscript, the MRC funder grant number (MR/S001751/1) was inadvertently omitted from the Funding section.

This error has been corrected.

