# Supplementary material for: Operative versus conservative management for inguinal hernia: a methodology scoping review of randomized controlled trials
Source: BJS Open. 2024 Sep 24;8(5):zrae116. doi: 10.1093/bjsopen/zrae116 (PMC11421466; doi:10.1093/bjsopen/zrae116)
Supplement: zrae116_Supplementary_Data [file zrae116_supplementary_data.zip › Supplementary_Material.docx]

## Operative versus conservative management for inguinal hernia: a methodology scoping review of randomised controlled trials

Maria Picciochi^1*^, Matthew J Lee^2*^, Samir Pathak^3^, Jessica Banks^4^, Jack A Helliwell^5^, Stephen J Chapman^4^ Neil Smart^6^, Katy Chalmers^7^, Sian Cousins^7^, Natalie Blencowe^3,7^

*****MP & MJL are joint first authors

**Affiliations:**

1 NIHR Global Surgery Unit, University of Birmingham, UK

2 Institute for Applied Health Research, University of Birmingham, UK

3 Department of Surgery, Leeds Teaching Hospitals NHS Trust

4 Division of Clinical Medicine, School of medicine and population health, University of Sheffield

5 Leeds Institute of Medical Research, University of Leeds, Leeds, UK

6 Department of Surgery, Royal Devon and Exeter Hospital

7 Centre for Surgical Research, University of Bristol, 39 Whatley Road, Clifton, Bristol BS8 2PS

**Corresponding author:** Natalie Blencowe, Natalie.blencowe@bristol.ac.uk

**Supplementary Materials - Index**

| **Supplementary Methods** |  |
| --- | --- |
| Search strategy | *Page 3* |
| **Supplementary Results** |  |
| Supplementary Table 1. All outcomes reported in studies | *pag. 4* |
| **Supplementary Figures and Tables** |  |
| Supplementary Figure 1. Median domain scores for PRECIS-2 assessments for each study | *pag. 5* |
|  |  |

**Supplementary Methods**

**Search strategy applied in MEDLINE/EMBASE**

**MEDLINE**  (run on 11/1/2023)

| 1 | exp Hernia, Inguinal/ |
| --- | --- |
| 2 | ((Inguina* or groin*) and herni*).mp. |
| 3 | 1 or 2 |
| 4 | exp Watchful waiting/ |
| 5 | active surveillance.mp. |
| 6 | Obser*.mp. |
| 7 | watch*.mp. |
| 8 | 4 or 5 or 6 or 7 |
| 9 | randomized controlled trial.pt. |
| 10 | controlled clinical trial.pt. |
| 11 | exp randomized controlled trial/ |
| 12 | exp controlled clinical trial/ |
| 13 | random*.tw. |
| 14 | clinical trial.sh. |
| 15 | trial.ti. |
| 16 | 9 or 10 or 11 or 12 or 13 or 14 or 15 |
| 17 | 3 and 8 and 16 |
| 18 | (child* or p*ediatric).ti. |
| 19 | 17 not 18 |

**Supplementary Results**

**Supplementary Table 1: All outcomes reported by each included study**

| **Outcome** | **Fitzgibbons** | **de Goede** | **O’Dwyer** |
| --- | --- | --- | --- |
| Deaths (not hernia related) | X |  | X |
| Pain | X | X | X |
| SF-36 | X | X | X |
| EQ-5D |  | X |  |
| Activities of daily living | X |  |  |
| Cost-effectiveness |  |  | X |
| ***Watchful waiting outcomes*** | | | |
| Acute incarceration | X | X | X |
| Crossover | X | X | X |
| ***Surgical outcomes*** | | | |
| Operative time |  | X |  |
| Conversion to open |  | X |  |
| Wound haematoma | X | X |  |
| Scrotal haematoma | X | X |  |
| Wound infection | X | X |  |
| Seroma | X | X |  |
| Ilioinguinal nerve injury | X |  |  |
| Urinary tract infection | X |  |  |
| Urinary retention |  | X |  |
| Ischaemic Orchitis | X | X |  |
| Post-operative hypertension | X |  |  |
| Deep vein thrombosis | X |  |  |
| Vessel injury |  | X |  |
| Bradycardia | X | X |  |
| Cardiac arrest |  | X |  |
| Hernia recurrence | X | X |  |
| Groin pain | X | X |  |
| Leg pain | X | X |  |
| Respiratory distress |  | X |  |
| Reoperation | X | X |  |
| Pain on ejaculation |  | X |  |

SF-36: Short Form Health Survey. EQ-5D: quality of life questionnaire evaluating 5 domains: Mobility, Usual Activities, Self-care, Pain & Discomfort and Anxiety & Depression.

**Supplementary Figures and Tables**

**Supplementary Figure 1: Median domain scores for PRECIS-2 assessments for each study**

|  | **PRECIS-2 domains** | | | | | | | | | |
| --- | --- | --- | --- | --- | --- | --- | --- | --- | --- | --- |
| **First author** | **Eligibility** | **Recruitment** | **Setting** | **Organisation** | **Flexibility: delivery** | **Flexibility: adherence** | **Follow-up** | **Primary outcome** | **Primary analyses** | **Mean score (trial)** |
| Fitzgibbons | 5 | 2 | 5 | NR | 2 | 3 | 2 | 5 | 5 | 4 |
| de Goede | 4 | 5 | 5 | NR | 5 | NR | 2 | 5 | 4 | 5 |
| O’Dwyer | 4 | NR | 4 | NR | 4 | NR | 3 | 5 | 5 | 4 |
| **Mean score (domain)** | 4 | 3.5 | 5 | NR | 4 | 3 | 2 | 5 | 5 |  |

Pragmatic

Equally pragmatic and explanatory

Explanatory

PRECIS: PRagmatic Explanatory Continuum Indicator Summary.
